# Early diagnosis of left ventricular diastolic dysfunction in diabetic patients: a possible role for natriuretic peptides

**DOI:** 10.1186/1475-2840-9-89

**Published:** 2010-12-16

**Authors:** Silvio Romano, Michele Di Mauro, Simona Fratini, Leonello Guarracini, Fabrizio Guarracini, Gianfranco Poccia, Maria Penco

**Affiliations:** 1 Cardiology, Department of Internal Medicine and Public Health, University of L'Aquila, Italy; 2 Cardiac Rehabilitation, "Umberto I" Hospital, Tagliacozzo, Italy; 3 Diabetology, "San Salvatore" Hospital, L'Aquila, Italy

## Abstract

**Background:**

The aim of the present study was to verify whether BNP might detect pre-clinical diastolic dysfunction (LVDD) in type-2 diabetic patients.

**Methods:**

One-hundred and twenty-seven consecutive outpatients with type-2 diabetes mellitus were enrolled into the study. Subjects with overt heart failure or NYHA class > 1, history of coronary artery disease, severe valvulopathy or chronic atrial fibrillation were excluded from the study. All patients underwent clinical evaluation, laboratory assessment of brain natriuretic peptide (BNP) and echocardiographic examination.

**Results:**

No patients showed systolic impairment of left ventricular function, whereas diastolic dysfunction was detected in 53 (42%) cases (all impaired relaxation). Median BNP was 27 pg/ml without any significant difference between 76 patients with normal left ventricular function and 53 with diastolic dysfunction; in 54 (43%) patients showing HBA1C≥8 (uncontrolled diabetes) normal function was found in 32 and diastolic dysfunction in 22, with a significant difference of BNP at multivariate analysis (OR = 1.02, 95%CI = 1.05-1.09, p = 0.003). In uncontrolled diabetic cohort, BNP was a strong predictor for LVDD (OR = 2.7, 95%CI = 1.3-5.6, p = 0.006) along with the duration of diabetes (OR = 1.6, 95%CI = 1.1-2.9, p = 0.046). BNP > 25 pg/ml was a cut-off value with high accuracy to detect a LVDD.

**Discussion:**

Early screening of high-risk patients for diabetic cardiomyopathy development might be useful to better control glycemic profile in order to reduce heart disease progression or even to reverse it

**Conclusions:**

BNP could be a cheap, easy and useful tool to screen those ones with preclinical ventricular diastolic dysfunction in a subset of patients particularly prone to develop cardiovascular complications, like uncontrolled diabetic patients.

## Background

Diabetes mellitus (DM) is not only a significant independent risk factor for developing of atherosclerotic ischemic heart disease or ventricular hypertrophy but it is also able to trigger a diabetic cardiomyopathy due to some dysmetabolic processes: inhibition of switching within the cardiomyocite from free-fatty acid (FFA) to glucose metabolism, dysregulation of FFA metabolism with increased uptake, reduced FFA oxidation, reduction of peroxisome proliferator-activated receptors (PPAR), increase of PPAR-γ and insulin-resistance, increased intracellular lipogenesis which leads to cardiomyocite lipotoxiocity [1].

The patients with type-2 DM are at 2-5 folds higher risk for developing heart failure (HF) [2]. Some large trials reported that the prevalence of diabetes in patients with chronic heart failure is around 30% and close to 50% in those with acute HF [3-5] The first stage of diabetic cardiomyopathy is represented by left ventricular diastolic dysfunction (LVDD) with preserved systolic function, in an asymptomatic pattern [6-9]; the prevalence of diastolic dysfunction largely ranges from 30% to 75%, depending on the echocardiographic parameters used to define it [7,10-13].

Internationally, there is an increased focus on prevention since even pre-clinical diabetic cardiomyopathy can impair significantly event-free survival [14], thus, in order to deal with this issue, neurohormonal profile of diabetic patients could be a good tool to screen subclinical ventricular dysfunction instead of cardiac imaging technique, which required elevated costs and human resource expenditure. For this purpose, brain natriuretic peptide (BNP) was demonstrated to be a good diagnostic and prognostic marker in diabetic patients with heart failure [15], systolic dysfunction [16], silent myocardial ischemia [17] and vascular complications [18]. The diagnostic role of natriuretic peptides for detecting LVDD in asymptomatic diabetic patients is still debated [14,17,19-21].

Hence, the aim of the present study is to verify whether BNP might detect pre-clinical diastolic dysfunction in type-2 diabetic patients.

## Methods

### Study population

One hundred and twenty-seven consecutive outpatients (age range 35-65 years, mean ± SD 55 ± 7 years), with type-2 diabetes mellitus according to ADA/WHO criteria [22] were enrolled into the study. Subjects with overt heart failure or NYHA class > 1, history of coronary artery disease, severe valvulopathy or chronic atrial fibrillation were excluded from the study.

All patients underwent clinical evaluation, laboratory assessment of BNP and echocardiographic examination (Table 1). Concerning antidiabetes therapy, 9% of them were on insulin treatment, 60% took oral antidiabetes agents and 6% had both treatments; the remaining 31% of patients were on diet. Other therapies were ACE-inhibitors (22%), ARB (9%), β-blockers (10%), calcium-channel blockers (6%), α-blockers (4%) and diuretics (12%).

**Table 1:** Demographic, clinical and echocardiographic characteristics.

POPULATION	OVERALL n = 127	Group A N = 74	Group B N = 53	p-value
Age (years)	55.2 ± 6.8	54.5 ± 6.8	56.3 ± 6.8	0.155

Gender (M/F)	65/62	38/36	24/29	0.500

BMI (Kg/m2)	29.2 ± 5.0	29.1 ± 5.1	29.5 ± 5.2	0.867

Diabetes duration (years)*	3 (1-10)*	3 (1-10)*	3 (1-9)	0.954

Smokers	28 (22%)	14 (19%)	14 (26%)	0.577

Ex-smokers	40 (31%)	25 (34%)	15 (28%)	0.504

Hypertension	51 (40%)	27 (37%)	24 (45%)	0.361

Hypercholesterolemia	29 (23%)	19 (26%)	10 (19%)	0.447

Creatinine (mg/dl)	0.89 ± 0.21	0.87 ± 0.21	0.94 ± 0.21	0.115

Microalbuminuria	34 (29%)	18 (28%)	16 (31%)	0.666

LVEF (%)	59.4 ± 4.8	60.5 ± 5.5	58.8 ± 3.7	0.143

LVMI (g/m2.7)	43.7 ± 11.8	42.6 ± 12.1	45.2 ± 11.3	0.198

LVH	51 (40%)	27 (36%)	24 (45%)	0.361

LVEF = left ventricular ejection fraction, LVMI = left ventricular mass index, LVH = left ventricular hypertrophy.

*data are expressed as median and interquartile range

All patients gave their written informed consent; the investigation conforms to the principles outlined in the Declaration of Helsinki and the study was approved by the local ethics committee (The University of L'Aquila).

### Laboratory

Blood samples were taken after 5 minutes of supine rest. BNP was measured using the commercially available AxSYM BNP assay, produced by Axis-Shield and Abbott Laboratories. Glycated hemoglobin (HBA1C) were measured using Menarini HA8140 assay. Microlabuminuria was defined as extemporary albuminuria value equal or greater than 20 mg/L.

### Echocardiography

M-mode, two-dimensional images and pulsed-wave and color-flow-Doppler examinations were performed on the same day of BNP assays, with a commercially available imaging system (ESAOTE MyLAB 30). Left ventricular systolic and diastolic volumes and ejection fraction (EF) were derived from the biplane apical modified Simpson's rule algorithm. Left ventricular dimensions were measured from M-mode images according to standard criteria. Diastolic dysfunction was assessed by means of transmitral pulsed-wave Doppler velocity (E/A ratio), deceleration time. In addition, pulmonary venous systolic and diastolic flow velocities were obtained as the maximal value reached during the corresponding phase of each cardiac cycle. Diastolic dysfunction was classified into 3 categories as follows: impaired relaxation, pseudonormal filling pattern and restrictive-like filling pattern, as previous reported [23]. Left ventricular mass (LVM) was calculated according to the following formula: 0.8 × [1.04 (IVS + LVDD + PWT)^3 ^- LVDD^3^] + 0.6 g [24]. All echocardiographic data were indexed by height to the allometric power of 2.7 [25] Left ventricular hypertrophy was defined as an LVM ≥ 50 g/m^2.7 ^in men and ≥ 47 g/m^2.7 ^in women.

A single observer (LG) made all measurements. Using digital archiving images, intraobserver variability was tested in a blinded fashion. Intraobserver variability was 3.3%, 3.9%, 5.1%, 8.5% and 9.2% for E/A ratio, E wave deceleration time, pulmonary venous systolic and diastolic flow velocities, EF and calculated left ventricular mass index (LVMI), respectively.

### End-points

The aim of this study was to evaluate the diagnostic role of BNP assays in early identification of diastolic dysfunction in diabetic patients, asymptomatic for heart failure. Particularly, since that an uncontrolled diabetes is strongly related to development of left ventricular dysfunction and chronic heart failure [21], a further analysis has been performed in this particular subset of patients. To define uncontrolled diabetes, an average of last two years HBA1C values were used; HBA1C equal or higher of 8% was chosen as threshold for uncontrolled diabetes, more prone to develop heart and vascular complications [26].

### Statistics

Results were expressed as mean (± standard deviation) for normally distributed continuous variables, whereas non-normally distributed data were expressed as median and interquartile range (IQR): 25^th ^and 75^th ^percentile; Categorical variables were reported as count and percentage. Distribution of continuous variables was tested for normal distribution using the Kolmogorov-Smirnov test. Paired-comparison was performed by means of Wilcoxon test whereas unpaired-comparison was performed by means of t-test or Mann-Whitney U test. Categorical data were compared by X2-test or Fisher exact test where appropriate. Logistic regression was used to identify predictive factors for LVDD. The variables initially inserted into the regression model were: age, gender, body mass index, systolic blood pressure, diastolic blood pressure, diabetes duration (years) smokers/ex-smokers, hypertension, hypercholesterolemia, obesity (BMI > 30 Kg/m2), left ventricular mass index (g/m2.7), last 2-year average HBA1C (%), creatinine (mg/dl), microalbuminuria, type of antidiabetic therapy, type of anti-hypertensive medication, BNP, hypertension plus left ventricular hypertrophy (duration of diabetes and BNP were non-normally distributed and so they underwent log-transformation before being inserted into the initially model). Variables included in the final model were reported as odd ratio (OR), 95% confidence interval (CI) and p-value.

Receiver operating characteristic (ROC) curve analysis was used to identify predictive cut-offs. Each statistical analysis was validated in 1000 bootstrap samples. A probability value p < 0.05 was considered significant. The retrospective power calculation, using as target end-point the comparison of BNP levels between uncontrolled diabetic patients with versus without diastolic dysfunction, estimates that 18 cases were the minimum sample size per each group to achieve a power of 80% with an α-error of 0.05.

All analyses were performed with the statistical software package SPSS (SPSS Inc, Chicago, IL, US).

## Results

No patients showed systolic impairment of left ventricular function, whereas diastolic dysfunction was detected in 53 (42%) cases; all of them were classified as impaired relaxation pattern; all the remaining 74 cases showed normal diastolic function. All the population had a creatinine equal or below 1.5 mg/dl; microalbuminuria was present in 29% of cases without any difference between patients with and without ILDD (Table 1)

**Figure 1:**
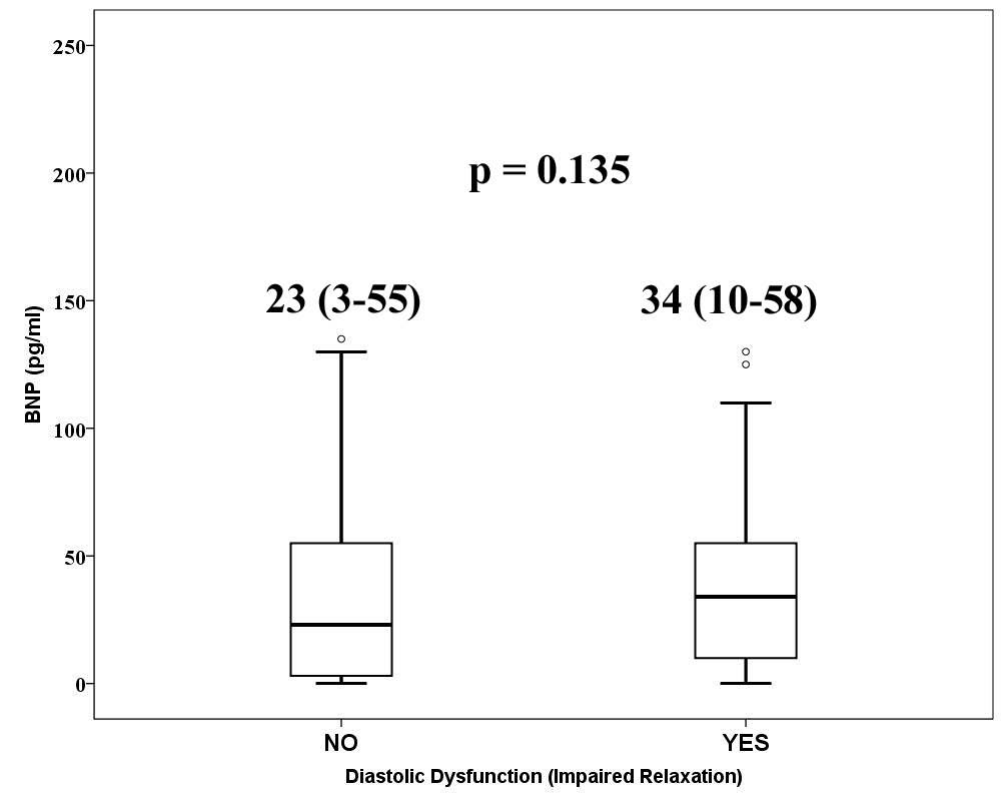
**Box-plots: BNP value according to absence or presence of diastolic dysfunction**. Numeric data reported in the figure are median and interquartile range.

Overall BNP median value was 27 pg/ml (IQR = 6-55) without any significant difference between 76 patients with normal left ventricular function (Group A) and those ones with diastolic dysfunction (Group B) (Figure 1). The only variable included into the final logistic regression model was the contemporary presence of hypertension and left ventricular hypertrophy (OR = 2.6, 95%CI = 1.1-6.5, p = 0.044).

Last two-year average HBA1C value was 7.7 ± 1.8 without any difference between the two groups (diastolic dysfunction 7.7 ± 1.8 vs normal function 7.7 ± 1.9, p = 0.905); Fifty-four (43%) out of 127 patients showed HBA1C≥8 (uncontrolled diabetes) with a mean value of 9.3 ± 1.7. The remaining group of patients (73) showed a mean HBA1C of 6.5 ± 0.6 (p vs uncontrolled diabetes < 0.001). In 73 patients with well-controlled diabetes, BNP level was not predictive for diastolic dysfunction (p = 0.244), whereas BNP seems to be a good predictive in the uncontrolled-diabetes cohort

The uncontrolled diabetes group was split into 2 groups: normal function (Group A, n = 32) and diastolic dysfunction (Group B, n = 22). The two groups did not show any difference concerning demographic, clinical, echocardiographic data (table 2).

**Table 2:** Difference between patient without and with diastolic dysfunction, in uncontrolled diabetic cohort (HBA1C≥8%).

POPULATION	Group A n = 32	Group B n = 22	p-value
Age (years)	52.6 ± 6.8	54.5 ± 8.4	0.355

Gender (M/F)	17/15	9/13	0.377

BMI (Kg/m2)	29.5 ± 5.1	30.2 ± 5.7	0.691

Diabetes duration (years)*	4 (0.7-10)	7 (3-10)	0.225

Smokers	6 (19%)	8 (36%)	0.303

Ex-smokers	8 (25%)	5 (23%)	0.848

Hypertension	7 (22%)	8 (36%)	0.243

Hypercholesterolemia	8 (25%)	5 (23%)	0.848

Creatinine (mg/dl)	0.90 ± 0.22	0.95 ± 0.24	0.407

Microalbuminuria	33 (33%)	30 (30%)	1.000

LVEF (%)	58.8 ± 5.2	58.9 ± 3.4	0.922

LVMI (g/m2.7)	38.7 ± 9.8	40.9 ± 11.4	0.461

LVH	4 (13%)	6 (27%)	0.170

HBA1C	9.3 ± 1.6	9.4 ± 1.8	0.707

Insulin	7 (22%)	4 (18%)	0.741

Oral antidiabetic agents	18 (56%)	15 (68%)	0.377

LVEF = left ventricular ejection fraction, LVMI = left ventricular mass index, LVH = left ventricular hypertrophy.

*data are expressed as median and interquartile range

**Figure 2:**
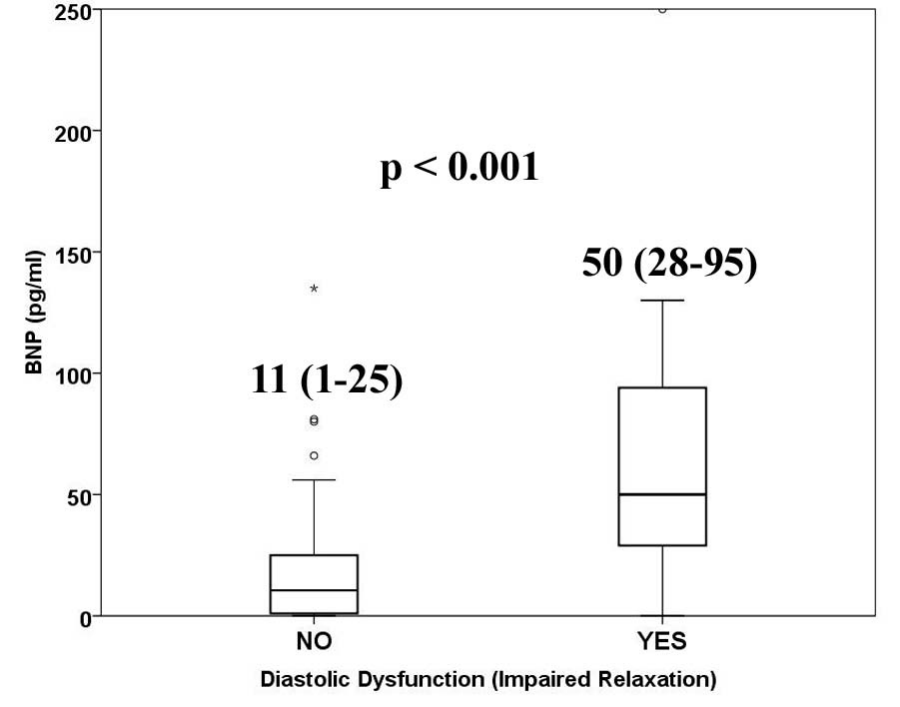
**Box-plots: BNP value according to absence or presence of diastolic dysfunction, in uncontrolled diabetic cohort**. Numeric data reported in the figure are median and interquartile range.

**Figure 3:**
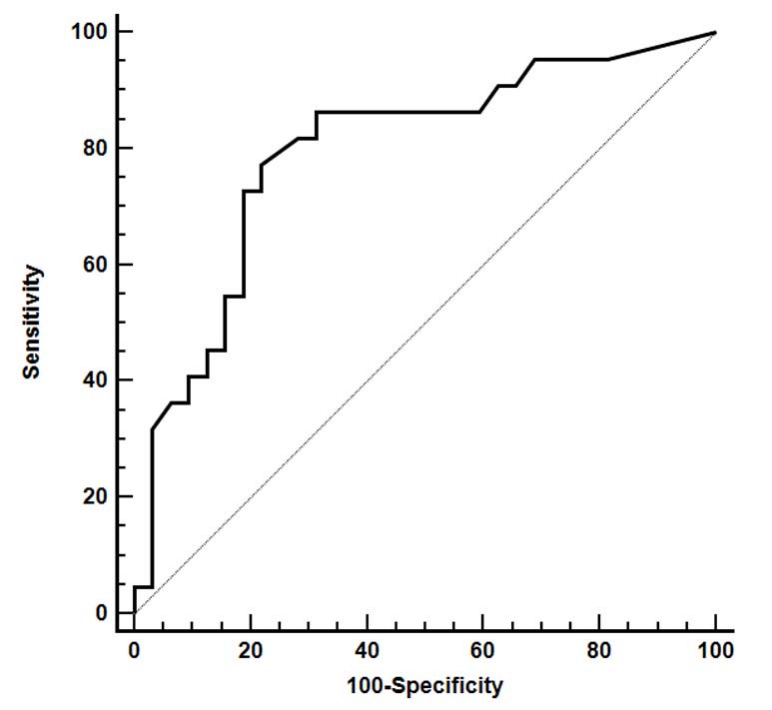
**ROC curve: BNP is a good predictor for DD in out of control cohort**.

The value of BNP was significantly higher in group B than in group A (Figure 2); the binary logistic regression confirmed that BNP was related to higher prevalence of LVDD (Log-transformation: OR = 2.7, 95%CI = 1.3-5.6, p = 0.006) along with the duration of diabetes (Log-transformation: OR = 1.6, 95%CI = 1.1-2.9, p = 0.046). Moreover, BNP was a strong predictor for LVDD in uncontrolled diabetes cohort (AUC = 0.80, 95%CI 0.66-0.89, p < 0.001) with a cut-off value of 25 pg/ml above that the likelihood to show a DD is high (sensitivity = 77% and specificity = 78%) (Figure 3); the rate of patients having BNP > 25 pg/ml was significantly higher in group B (77% vs 22%, p < 0.001).

## Discussion

### Prevalence of subclinical isolated diastolic dysfunction in type-2 diabetic population

Our data show a prevalence of asymptomatic diastolic dysfunction of roughly 42%, perfectly included within the range, from 30% to 63%, reported by those studies where LVDD was assessed by means of conventional echocardiography [7,10-13,27,28]. In our series, the presence of LVDD was independent by age and gender unlike reported by other Authors which clearly found a relationship between these factors and the prevalence of LVDD in diabetic patients [7,10,11]. Anyway, the presence of hypertension along with left ventricular hypertrophy increased the prevalence of LVDD up to 61% (14/23) versus 38% (39/104), p = 0.040; this result was confirmed also by binary logistic regression (OR = 2.6, 95%CI = 1.1-6.5, p = 0.044), corroborating that combination of DM and HTN has more severe impact on ventricular filling pattern [27].

However, the diastolic function was impaired also in 38% of 104 diabetic patients without HTN+LVH, similar either in presence of HTN or not (p = 0.819). To explain the patho-physiological basis of this left ventricular impaired relaxation, the concept that diabetes per se might cause a stand-alone cardiomyopathy should be accepted [1]. Diabetic cardiomyopathy has been defined as the presence of myocardial abnormalities in the absence of coronary artery disease, hypertension or other significant etiology [29] In a very recent report, Anderson and coworkers [30] compared two cohorts of 31 patients having EF > 35%, without significant coronary artery disease (CAD), prior myocardial infarction, cardiac pacemaker, atrial fibrillation, or significant valve disease, one diabetic and the other one controls, matching the two groups on age, gender and presence of hypertension. The Authors concluded that In this set of patients diabetes is anyway associated with global diastolic dysfunction. This finding is in accordance with the hypotheses of increased myocardial stiffness, increased resting myocyte tension and deposition of advanced glycated end products associated with diabetic cardiomyopathy; In fact, intracellular hyperglycemia is at basis of formation of advanced-glycated end products (AGEs) as collagen, elastin and other connective tissue proteins [31] which produce myocardial fibrosis resulting in diastolic dysfunction.

### BNP and diastolic dysfunction

The diagnostic role played by BNP in early detection of asymptomatic diastolic dysfunction in type-2 diabetes is still controversial [14,16,19-21,32-34]. In our, series, we failed to identify any difference between patients with normal function and those ones with LVDD regarding BNP value (Figure 1). The possible explanation of this results can be the presence of just impaired relaxation in our population which represents the mild grade of LVDD [19,20]. In fact, Magnusson et al, [19] described a detecting role of BNP with respect to LVDD, but only in case of moderate-to-severe LVDD; comparing patients with and without mild LVDD the Authors did not find any difference (lnBNP 1.3 ± 0.8 vs 0.98 ± 0.85, p = 0.09) and this finding was confirmed also at multivariate analysis. Conversely, comparing patients with moderate-to-severe LVDD, lnBNP was significantly higher (2.1 ± 1.4 vs 0.98 ± 0.85, ' < 0.0001). Kiencke and co-workers [14] analyzed 100 adults with diabetes in order to evaluate if BNP could predict pre-clinical diastolic dysfunction in diabetic cardiomyopathy. They concluded that BNP had higher prognostic than diagnostic role. Although the ROC curve analysis identified BNP as predictor for diastolic dysfunction, the area under curve (0.70) showed a low accuracy of BNP test and the identified cut-off value of 34 pg/dl showed a moderate sensitivity (66%) and specificity (71%). The suggestion that BNP is very likely unable for early detection of LVDD was confirmed by others [20].

On the contrary, Albertini and co-workers [16] assessed BNP value in 91 consecutive patients with type 2 diabetes mellitus, finding that BNP level was significantly higher in patients with LVDD, especially in case of untreated hypertension (87 ± 20 vs 13 ± 2, p < 0.0001); anyway, comparing the mean value of patient with normal LV function with those ones with LVDD, independently from the anti-hypertensive treatment, BNP was significantly higher in the latter group (p < 0.001). In another report, the area under the receiver-operating characteristic curve for NT-proBNP to separate normal versus diastolic dysfunction, in type 2 diabetic patients, was even 0.96 [32]. Kim et al [33] assessed NT-proBNP levels in a group consisted of 130 diabetic patients referred for echocardiography, finding that in diastolic dysfunction were significantly higher than normal group (1491.1 pg/mL versus 232.3 pg/mL, P = 0.01), even though there was no difference in ejection fraction (EF) (61.2+/-7.9% versus 60+/-8.4%, P = 0.773).

### BNP and diastolic dysfunction in uncontrolled diabetic subset

Also Dencker et al [21] demonstrated that BNP was significantly higher in patients with abnormal diastolic dysfunction (26.0 ± 3.4 vs 5.3 ± 3.4, p < 0.001) but in a cohort of 33 patients with poorly regulated type 2 diabetes. The authors defined "poorly regulated" when HBA1C > 7%. In 2001, Menzin J et al [26] found that HBA1C <8% had roughly 60% fewer hospital admissions for complications, especially heart complications. Thus, we decided to perform a sub-analysis in 54 patients with uncontrolled diabetes (HBA1C≥8%), in order to verify if BNP might be a good tool for early detection of LVDD at least in this subset of patients. Both univariate and multivariate analysis confirmed that higher levels of BNP were associated with LVDD and ROC curve analysis demonstrated the good accuracy of this parameter for detecting LVDD; furthermore, a cut-off value was also identified BNP> 25 pg/ml, with a good sensitivity (77%) and specificity (78%) (Figure 3). This finding is consistent with the one reported by Albertini et al [16]. Conversely, in 73 patients with well-controlled diabetes, BNP level was not predictive for diastolic dysfunction.

### Limitations of the study

The main limitation of this study is the small sample size. Another important limits is related to the echocardiographic assessment of diastolic dysfunction, that, nowadays, is more and more evaluated by means of tissue Doppler imaging (TDI).

## Clinical implications and Conclusion

There is an important concept raising up from the present study, BNP is a good marker for detecting pre-clinical LVDD in a subset of patients particularly prone to develop cardiovascular complications, like those ones with uncontrolled diabetes. In clinical practice it is very difficult to submit all asymptomatic diabetic subjects to echocardiographic lab; so BNP could be a cheap, easy and useful tool to screen those ones with preclinical ventricular diastolic dysfunction among patients with high HBA1C. Furthermore, BNP could be use for serial assessment of this specific cohort of patients asymptomatic for heart failure.

Another clinical implication concerns the possibility to better control glycemic profile in order to reduce heart disease progression or even to reverse it, especially in patients with short duration of diabetes without history of cardiovascular event [35,36]; In fact, in the early stages of diabetes, structural modifications seem to be partially reversible [8]. Finally, early screening of those patients with subclinical diastolic dysfunction, is useful to plan a cardiologic therapy to prevent the development of diabetic cardiomyopathy.

## Competing interests

The authors declare that they have no competing interests.

## Authors' contributions

MP and LG conceived and designed the study protocol, moreover gave their final approval of the manuscript. LG performed echocardiography. SF, FG, GP provided collection of data. SR and MDM performed analyses and prepared the manuscript. All authors have read and approved the manuscript.
